# Different creep compound feed formulations for new born piglets: influence on growth performance and health parameters

**DOI:** 10.3389/fvets.2022.971783

**Published:** 2022-08-29

**Authors:** Sarunas Badaras, Modestas Ruzauskas, Romas Gruzauskas, Egle Zokaityte, Vytaute Starkute, Dovile Klupsaite, Ernestas Mockus, Jolita Klementaviciute, Laurynas Vadopalas, Gintare Zokaityte, Agila Dauksiene, Vadims Bartkevics, Elena Bartkiene

**Affiliations:** ^1^Institute of Animal Rearing Technologies, Faculty of Animal Sciences, Lithuanian University of Health Sciences, Kaunas, Lithuania; ^2^Institute of Microbiology and Virology, Faculty of Veterinary Medicine, Lithuanian University of Health Sciences, Kaunas, Lithuania; ^3^Department of Anatomy and Physiology, Faculty of Veterinary Medicine, Lithuanian University of Health Sciences, Kaunas, Lithuania; ^4^Department of Food Science and Technology, Kaunas University of Technology, Kaunas, Lithuania; ^5^Department of Food Safety and Quality, Faculty of Veterinary Medicine, Lithuanian University of Health Sciences, Kaunas, Lithuania; ^6^Institute of Food Safety, Animal Health and Environment BIOR, Riga, Latvia

**Keywords:** creep compound feed, piglets, wheat bran, sugar beet pulp, microbial profile, health, growth performance

## Abstract

The aim of this study was to compare the influence of different compositions of creep compound feed (CCF) (C-I – control group; TG-II – a CCF containing wheat bran extruded and fermented with *L. paracasei*; TG-III – a creep compound feed containing sugar beet pulp) on the piglets' growth performance, blood parameters, fecal microbial profile and physicochemical characteristics. Moreover, the fecal volatile compound (VC) profile was analyzed as a possible chemical marker related to changes in the fecal microbial profile and physicochemical characteristics. A 21-day experiment was conducted using 1-day-old 300 Large White/Norwegian Landrace piglets. The highest body weight (at the 21st day) was found in piglets of the TG-III group, and both treated groups showed lower feed conversion ratios. At the end of the experiment, significantly higher *lactobacillus* counts in the feces of both treated groups were found, and a correlation between fecal textural hardness and the *lactobacillus* count was established (*r* = 0.475). Significant correlations of piglets' individual fecal VC with microbiological parameters and fecal pH were established [*lactobacilli* with 3-n-nonadecanol-1; enterobacteria with butyric acid <2-methyl->; pentanoic acid, 4-methyl-; eicosene(E)-, etc.]. It can be concluded that local material could be successfully incorporated into CCF preparation without impairing animal metabolism.

## Introduction

An effective method of increasing the sustainability of the livestock chain is to include more local material for animal nutrition, with the aim of reducing exports and increasing the stability of the local industry by reducing its dependence on the import of raw materials. Feed production and the feed base are the bases for the sustainable development of highly productive animal husbandry In addition, feed production is especially important for the sustainable use of local agricultural resources, the reduction of problems related to undesirable climate changes and the overall public satisfaction of having an opportunity to choose local products prepared by using local stock.

The food industry generates large amounts of different valuable by-products, which have been undervalued until now. As an example, to date, wheat processing by-products, have been used without any pre-treatment as a feed stock of low nutritional value; however, their functional value could be increased and their safety characteristics improved (1). It was reported that, in Europe, more than 45 million tons/year of wheat and oats are processed, generating more than 6.5 million tons of bran that are further used for the livestock industry (2). Cereal brans are an abundant source of valuable compounds; in addition, their pre-treatment could increase their functional value and safety characteristics (1). Our previous studies showed that the nutritional and functional value of wheat bran (WB) as well as its safety parameters could be improved by using combined extrusion and fermentation technology under optimized conditions [selected extrusion parameters and the lactic acid bacteria (LAB) strain used for fermentation] (1). Also, during the extrusion and fermentation processes, the mycotoxin concentration in WB is reduced, and this parameter is key, especially when further material is used for piglet nutrition, as piglets are very sensitive to low mycotoxin concentrations in feed. Our previous studies also showed that feed fermented with selected LAB strains could positively affect the piglets' microbiota and lead to better detoxification of mycotoxins *in vivo* (3). Also, it was reported, that the abundance of bifidobacteria in fecal microbiota was significantly higher for weaned piglets fed the WB diet than the piglets fed with other dietary fiber rich sources, because of WB arabinoxylans (4).

In this study, we hypothesized that extruded and fermented WB, when included in the composition of creep compound feed (CCF), could lead to desirable changes in the microbiota of piglet feces, which could lead to better health parameters and growth performance.

Another raw material that could be obtained locally is sugar beet pulp. The latter could be a valuable ingredient in CCF for piglets. It was reported that sugar beet fiber consists, on average, of 29% hemicelluloses, 23% cellulose, 22.3% uronic acids, ~1–2% lignin, ~7–8% protein, 7.5–12% ash and ~0.5% residual sucrose. Previous studies showed that the inclusion of 12% sugar beet pulp in weaned piglets' diets can improve their health status and growth performance (5). However, the abovementioned study does not provide clear evidence of these positive changes. It was reported, that the sugar beet pulp supplementation increased *Lactobacillus* counts in piglets feces (6). These desirable changes were explained, by the influence of sugar beet pulp soluble dietary fiber compounds, which increasing the number and activity of microbes in the large intestine, and even in the ileum, as well as a reduction in pH, which accelerating growth of beneficial bacteria, i.e., *Lactobacillus* (7). In this study we hypothesized that the positive influence on piglet health could be related to desirable changes in the microbial composition of the digestive tract, which has a further positive influence on the main health parameters and growth performance.

Finally, piglet weaning under industrial conditions involves many stressors, including a change in diet from sow's milk to solid feed. Reducing the nutritional stressors at weaning may help to mitigate weaning-related problems by habituating piglets to solid feed as an alternative energy source and may reduce neophobia for the weaner diet. The early-life microbial colonization is the most important time for shaping intestinal and immune development (8). Piglets face many early-life events that shape the acquisition and development of their intestinal microbiota, as well as CCF could be one from the factors, which can be involved in this process.

The aim of this study was to compare the influences of different compositions of CCF on the piglets' growth performance, blood parameters, fecal microbial profile and physicochemical characteristics. In addition, fecal volatile compounds were identified as potential chemical markers related to changes in the fecal microbial profile and physicochemical characteristics.

## Materials and methods

### Material used for piglet feed preparation from creep compound

Processed (extruded and fermented) WB as obtained from the SME “Ustukiu malunas” (Pasvalys, Lithuania). Physico-chemical and microbiological characteristics of the extruded and fermented WB were reported by Zokaityte et al. (1). For piglet feeding (to minimize the mycotoxin content (W_ex130/screwspeed25Lpa_), were selected. Physicochemical, antimicrobial, and antifungal characteristics of the W_ex130/screwspeed25Lpa_ are given in Supplementary material 1.

Sugar beet pulp (dried) was obtained from the company Imlitex Agro (Kaunas, Lithuania). The composition of sugar beet pulp was moisture – 12.0%, crude protein – 10.4%, crude fat – 0.850%, crude fiber – 17.8%, calcium – 1.34% and phosphorus – 0.120%.

### Animals and housing

The study was conducted on a pig farm in the Silales district (Misuciai, Lithuania) and at the Institute of Animal Rearing Technologies, Lithuanian University of Health Sciences (Kaunas, Lithuania). A 21-day experiment was conducted using 300 1-day-old Large White / Norwegian Landrace (LW/NL) piglets (100 piglets in each group). The farrowing crates (containing a heated creep mat for piglets) were 4.32 m^2^ (1.8 × 2.4 m), of which 0.15 m^2^ per piglet and 1.76 m^2^ per sow. The control and trial groups contained 10 replications. In one box was one sow with 10 suckling piglets. The groups were formed on an analog basis. The sows were fed with compound feed [in accordance with NRC (2012) requirements]. Drinking water was available *ad libitum* throughout the trial. The trial started with piglets at an initial body weight of 1.76–1.81 kg in all (control and both treatment) groups. The piglet's diet before the trial was composed of 20.0% crude protein, 3.01% crude fiber, 6.26% crude fats, 1.50% av. lysine, 0.610% av. methionine, 0.320% av. tryptophan, 0.940% av. threonine, 0.800% Ca and 0.400% av. P. Antibiotic treatment was not applied.

### Experimental design and diets

The piglets were distributed into three groups. Three dietary treatments were compared: (i) control CCF (C-I – control group), (ii) CCF containing W_ex130/screwspeed25Lpa_ (TG-II) and (iii) CCF containing sugar beet pulp (TG-III). All animal groups were fed with wet feed (water and feed in a 3:1 ratio), and the equipment used for feeding was WEDA (Dammann & Westerkamp GmbH, Germany). The amount of CCF in the feed trough was checked at least twice daily to provide the CCF *ad libitum*, and all CCF was replaced at each feed weigh-back to maintain the freshness of the feed.

The piglets' growth performance was evaluated by testing all 100 piglets from each group; microbiological feces parameters were analyzed by testing 40 piglets from each group, and other piglet parameters were evaluated by testing 10 piglets from each group. The basal feed was formulated according to the nutritional requirements described in (9). The feed composition and nutritional values are shown in Table 1. Dietary contents were analyzed according to the AOAC recommendations (10).

**Table 1:** Composition of control and experimental piglet diets.

**Ingredients (%)**	**C-I**	**TG-II**	**TG-III**
Barley	8.00	10.0	8.0
Wheat	31.0	24.2	28.0
Extruded Wheat	18.0	20.0	18.0
Extruded + fermented wheat bran	–	3.0	–
Sugar beet pulp, dry	–	–	3
Fermented soybean meal	5.0	5.0	5.0
Potato protein	1.5	1.5	1.5
Soybean protein concentrate	8.0	8.0	8.0
Whey powder	10.6	10.6	10.6
Lactose	4.0	4.0	4.0
Egg powder	3.0	3.0	3.0
Soybean oil	5.0	5.0	5.0
MCFA (C8:0–25%; C10:0–25%, C12:0–30%)	0.2	0.2	0.2
Calcium butyrate	0.2	0.1	0.2
Limestone	1.00	1.0	1.0
NaCl	0.6	0.6	0.6
Monocalcium phosphate	0.4	0.3	0.4
L-Lysine sulfate, L-Lysine 54.6%	1.3	1.3	1.3
DL-Methionine	0.4	0.4	0.4
L-Threonine	0.4	0.4	0.4
L-Tryptophan	0.2	0.2	0.2
Acidal NC (formic and acetic acids)	0.2	0.2	0.2
^1^Vitamins and trace elements (premix)	1.0	1.0	1.0
**Nutritional value**			
ME swine (MJ/kg)	13.9	13.8	13.8
Crude protein (%)	20.0	19.9	19.9
Crude fat (%)	7.8	7.8	7.8
Crude fiber (%)	2.5	2.6	2.9
Lysine (%)	1.7	1.7	1.7
Methionine (%)	0.7	0.7	0.7
Threonine (%)	1.1	1.1	1.1
Tryptophan (%)	0.4	0.4	0.4
Methionine + Cystine (%)	1.0	1.0	1.0
Ca (%)	0.8	0.8	0.8
Total P (%)	0.6	0.6	0.6
Available P (%)	0.4	0.4	0.4
Na (%)	0.3	0.3	0.3

(i) basal diet (C-I – control group), (ii) basal diet supplemented with wheat bran extruded at 130°C and 25 rpm and fermented with *Lactobacillus paracasei* (TG-II), (iii) basal diet supplemented with dried sugar beet pulp (TG-III).

1 Composition of premix per 1 kg of feed: Vitamin A-18.180 IU; vitamin D3-2.000 IU; vitamin E-160 mg/kg; vitamin K3-5.00 mg; thiamine-3.64 mg; riboflavin-9.16 mg; choline chloride-4 mg; pyridoxine-4.60 mg; vitamin B12-0.05 mg; niacin-40.54 mg; pantothenic acid-22.54 mg; folic acid-1.80 mg; biotin-0.2 mg; Fe-150 mg; Cu-101 mg; Zn-100 mg; Mn-84 mg; I-0.72 mg; Co-0.50 mg; Se-0.40 mg. For all groups, the following were added to the compound feed: NSP Enzyme, Rovabio Excel AP, 50 g/t; endo-1,4-β-xylanase 1 100 VU /kg of feed; endo-1,3 (4)-glucanase, 1,500 VU /kg of feed and Phytase Axtra PHY 10000 TPT 2, 130 g/t, 1,300 FTU/kg feed.

### Evaluation of piglets' growth performance

Group body weight (BW) gain was recorded on days 1, 10, 17, and 21 of age using an electronic weighing system (model type: IT1000, SysTec GmbH Bergheim, Germany). The feed conversion ratio (FCR) was calculated from feed intake (87% of dry matter) and BW gain, which was recorded on the same days as BW gain using a WEDA (Dammann & Westerkamp GmbH, Germany) automated feeding system that has an electronic flowmeter and weighing system.

### Blood analysis

Piglets were bled from the jugular vein into vacuum blood tubes (BD Vacutainer, United Kingdom) before the morning feeding. Tubes with clot activator were used for biochemical examination. To avoid stressing new-born piglets, blood biochemical variables were evaluated after the experiment on day 18 of the piglet's life. The parameters included aspartate aminotransferase (AST), alanine aminotransferase (ALT), cholesterol (CHOL) (mmol), high-density lipoprotein cholesterol (HDL-C), low-density lipoprotein (LDL-C) cholesterol, triglycerides (TG), total protein (TP), albumin (ALB), triiodothyronine (T3), thyroxine (T4), immunoglobulins IgA, IgM and IgG, glucose (GLU), creatinine analyzed by the Jaffe method (CREA), alkaline phosphatase (AP), thyroid-stimulating hormone (TSH), total bilirubin and urea. Blood parameters were analyzed with an automatic biochemistry analyser in the accredited laboratory “Anteja” (Klaipeda, Lithuania).

### Evaluation of fecal pH, dry matter and color characteristics

The fecal pH was analyzed with a pH meter (Inolab 3, Hanna Instruments, Italy). The dry matter (DM) of the feces was evaluated after drying the samples at 103 ± 2°C to a constant weight. The color coordinates were measured at three different points on the fecal sample surface using the CIE L^*^a^*^b^*^ system (CromaMeter CR-400, Konica Minolta, Tokyo, Japan).

### Analysis of the fecal volatile compound profile

Feces were prepared for gas chromatography (GC) analysis by using solid-phase microextraction (SPME). An SPME device with Stableflex (TM) fiber, coated with a 50-μm DVB-PDMS-Carboxen™ layer (Supelco, USA), was used for sample preparation. For gas chromatography–mass spectrometry (GC-MS), a GCMS-QP2010 (Shimadzu, Japan) was used. The gas chromatograph was equipped with an AOC-5000 Plus Shimadzu autosampler, upgraded with an SPME analysis kit. Analysis was performed according to the procedure described by Vadopalas et al. (11).

### Microbiological analysis of fecal samples

Fecal samples were collected from 40 piglets from each group before (at day 1) and after the experiment (at day 21), stored in vials (+4°C) with transport medium (*Faecal Enteric* Plus, Oxoid, Basingstoke, UK) and analyzed on the same day. Evaluation of microbiological parameters [LAB, total bacteria count (TBC), total enterobacteria count (TEC), *Enterococcus faecalis* count and yeast and mold counts (Y/M)] was performed according to methods described by Zavistanaviciute et al. (12).

### Microbial profiling analysis

Before the piglets were divided into groups, feces from 10 healthy piglets (from each group) were taken, and a single pooled sample was prepared for microbiome profiling using next generation targeted sequencing of 16S rRNA. After the experiment, feces from 10 piglets from the control and both treated groups were collected (30 samples in total), and thereafter, three pooled samples representing each of the groups were prepared. Samples were stored in DNA/RNA Shield (1:10 dilution; R1100-250, Zymo Research, USA) at −70°C until DNA extraction. DNA was extracted with a fecal DNA MiniPrep kit (D6010, Zymo Research, USA). Metagenomic libraries were prepared, sequenced, quality controlled, and assembled in an independent service laboratory (Baseclear, the Netherlands). Short paired sequence reads were generated using the Illumina MiSeq system (Illumina, USA) and converted into FASTQ files using the BCL2FASTQ pipeline software, version 1.8.3 (Illumina). Quality trimming was applied based on Phred quality scores. Subsequently, the Illumina paired reads were merged into single reads (so-called “pseudoreads”) through sequence overlap. Chimeric pseudoreads were removed, and the remaining reads were aligned to a combination of the GreenGenes and RDP 16S gene databases. Based on the alignment scores of the pseudoreads, the taxonomic classes were assigned by associating each pseudoread to the best matching Operational Taxonomic Unit (OTU). ZymoBIOMICS Microbial Community Standard (D6300, Zymo Research, Murphy Ave. Irvine, CA, USA) was used as a microbiome profiling quality control. The results of taxonomic classification were visualized using the interactive online platform (https://genome-explorer.com).

### Ethical statement

All animal procedures were conducted according to the EU Directive of the European Parliament and of the Council from September 22, 2010 (13) on the protection of animals used for scientific purposes and Requirements for the Keeping, Maintenance, and Use of Animals Intended for Science and Education Purposes, approved by the order of the Lithuanian Director of the State Food and Veterinary Service (14). Research was carried out in accordance with the Republic of Lithuania Act (15).

### Statistical analysis

In order to compare the influence of different compositions of creep compound on the fecal microbial profile and physicochemical characteristics, blood parameters and growth performance in piglets, multivariate general linear model (GLM) was used for data analysis (SPSS for Windows, Ver.15.0, SPSS, Chicago, Illinois, USA). The *p*-values of factors interaction (experiment day × treatment type) were determined by tests of between-subjects effects in multivariate GLM. Baseline measurements were used as covariates to account for the experimental conditions. The mean values were compared using Duncan's multiple range *post-hoc* test with the significance level defined at *p* ≤ 0.05. In the tables, the results are presented as mean values with pooled standard errors. Pearson correlations were also calculated; correlations were considered significant when *p* ≤ 0.05. Differences in bacterial genera between the groups were assessed using the Z-test calculator for two population proportions (Social Science Statistics, socscistatistics.com, 2019). The number of reads of each genera from the total number of reads in samples was counted. Relative abundance of the genera in samples then were compared between the groups. Two-tailed hypothesis was counted. For the quality control of taxonomical identification, standard (D6300, Zymo Research, Murphy Ave. Irvine, CA, USA) of mixed known bacterial cultures were sequenced together with sample sequencing. Statistical comparisons were considered significant when *p* ≤ 0.05.

## Results and discussion

### Piglets' growth performance results

**Figure 1:**
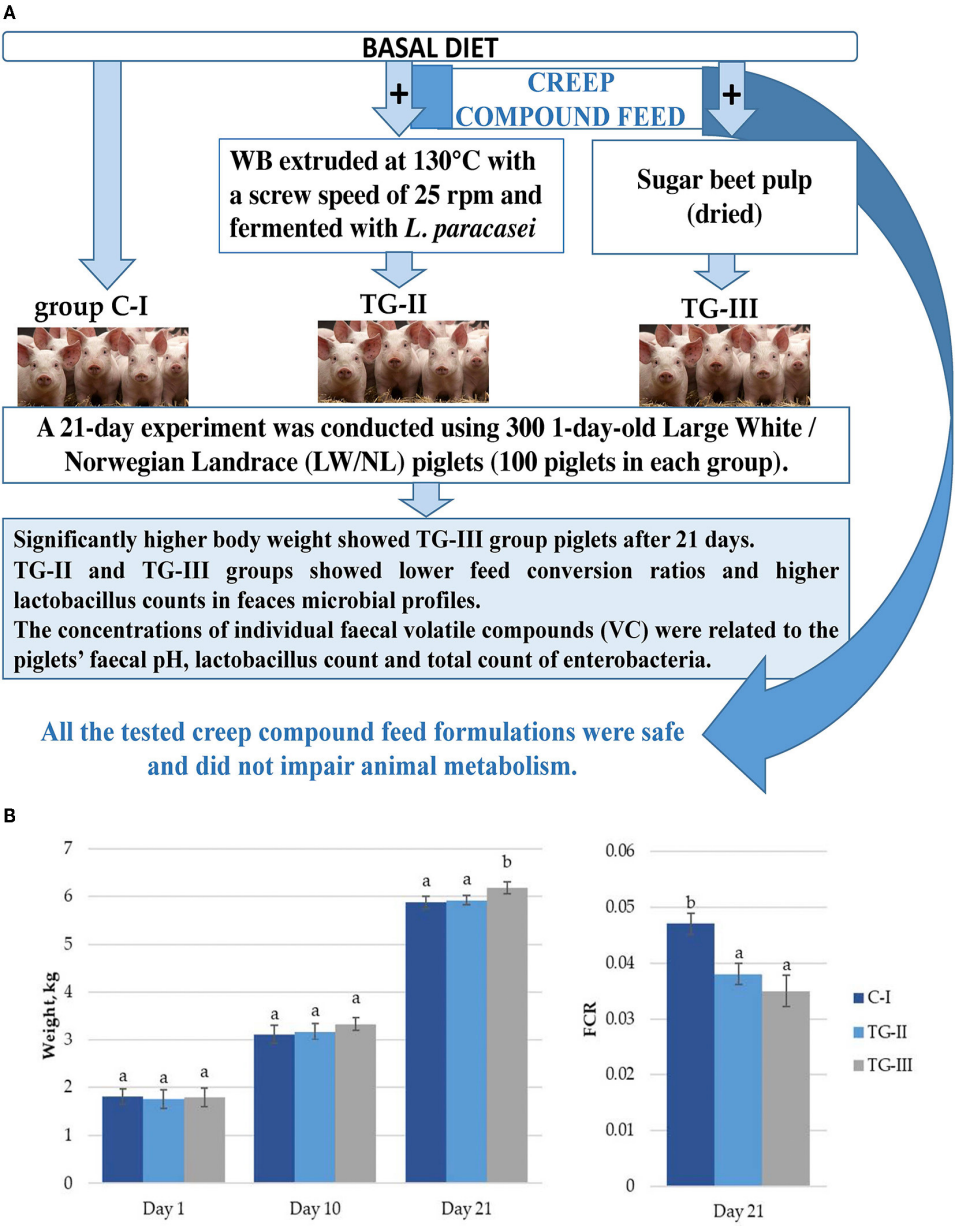
**(A)** Piglet weight; **(B)** Feed conversion ratio (FCR) (^a, b^different letters indicate differences among treatments (*p* < 0.05); (i) basal diet (C-I – control group), (ii) basal diet with extruded-fermented wheat bran added (TG-II), (iii) basal diet with dried sugar beet pulp (TG-III). The data are presented as the mean ± standard error (*n* = 100/group).

In a comparison of body weight (BW) gain in piglets, significantly higher BW in piglets of the TG-III group at 21 days was established (Figure 1A). By comparison, the feed conversion ratio (FCR) after 21 days in both treated groups was lower, in comparison with the C-I group (Figure 1B). Piglet mortality was 7.02%, in the control (CI) group, 5.19% in the TG-II group and 2.92% in the TG-III group. Beet pulp has high levels of crude fiber (CF) (including a large amount of the polymer l-arabinose), nitrogen-free leachate and crude protein (CP) (16). CF serves as the main fermentation substrate, particularly for microorganisms in the hindgut of piglets (17). The beneficial effect of sugar beet addition on BW and the FCR could be mainly attributed to alteration of the bacterial composition in the digestive tract, especially *Lactobacillus* sp., and the short-chain fatty-acid (SCFA) acetate, propionate and butyrate contents. Moreover, the intestinal morphology is also affected by dietary fiber, and increased intestinal villus height and the ratio of villus height to crypt depth influences intestinal nutrient absorption capacity in pigs, thus leading to increased BW.

Variable findings are presented in the literature regarding the impact of dietary sugar beet on productivity in piglets. In contrast, Lizardo et al. reported that addition of sugar beet in the diet had a positive effect on growth performance in weaning pigs (18).

### Blood parameters

The blood parameters of 18-day-old piglets are given in Table 2. Significant differences in the immunoglobulins IgA and IgM concentrations between blood samples of different groups of piglets were not found. However, the lowest IgG concentration showed TG-III group samples and the treatment was not significant factor on all the tested immunoglobulins concentration. Blood serum characteristics are related to the animal's health and nutritional status and could reveal the possible interaction between bacteria and the host's metabolism. Wheat bran is rich in fiber, minerals, B vitamins and lot of bioactive and volatile compounds with health benefits (19). Chemical composition of sugar beet pulp consist mainly of cellulose, hemicellulose and pectin (20). It is known that non-digested fibers may serve as substrate for beneficial bacteria, altering the gut microbiota and increasing the proportion of lactic bacteria (21). The antibodies IgA, IgG, and IgM antibody play a crucial role in immune response of piglets to disease infection (22). Silva et al. reported, that the concentrations of blood IgA values were lower in piglets consuming cellulose (23). This can be explained by the lower incidence of diarrhea, considering that the production of immunoglobulins is stimulated by enteric infections of the gut mucosa (23).

**Table 2:** Blood parameters of 18-day-old piglets.

**Blood parameters**	**Treatments**			***p***			
	**C-I**	**TG-II**	**TG-III**	**C-I × TG-II**	**C-I × TG-III**	**TG-II × TG-III**	**Significance of the analyzed factor (diet)**
Immunoglobulin (IgA), g/L	0.32 ± 0.011	0.32 ± 0.010	0.26 ± 0.081	0.500	0.306	0.317	0.279
Immunoglobulin (IgM), g/L	0.21 ± 0.005	0.2 ± 0.012	0.17 ± 0.094	0.317	0.309	0.308	0.667
Immunoglobulin (IgG), g/L	3.23 ± 0.748 b	3.35 ± 1.441 b	1.92 ± 1.316 a	0.395	0.029	0.001	0.340
Thyroid-stimulating hormone (TSH), μIU/mL	0.017 ± 0.002 b	0.02 ± 0.001 c	0.01 ± 0.002 a	0.034	<.001	0.006	0.002
Albumin (ALB), g/L	32.4 ± 1.673	32.6 ± 0.548	30.8 ± 5.805	0.394	0.286	0.307	0.795
Total protein (TP), g/L	35.6 ± 11.768 a	44.0 ± 2.549 b	41.6 ± 3.159 b	0.017	0.039	0.085	0.410
Urea, mmol/L	1.92 ± 0.268 a	2.0 ± 0.292 b	1.88 ± 0.1304 a	0.013	0.333	0.163	0.829
Creatinine (CREA), μmol/L	58.02 ± 8.067 b	61.88 ± 4.003 b	55.58 ± 9.587 a	0.121	0.044	0.095	0.617
Alanine aminotransferase (ALT), U/L	41.2 ± 2.698 a	49.0 ± 6.483 b	46.6 ± 9.788 b	0.032	0.127	0.107	0.469
Aspartate aminotransferase (AST), U/L	33.8 ± 5.016 a	37.0 ± 3.391 b	34.0 ± 8.916 a	0.038	0.469	0.223	0.803
Alkaline phosphatase (AP), U/L	1,077.2 ± 313.56 b	907.2 ± 127.92 a	941.8 ± 323.61 a	0.127	0.001	0.394	0.730
Total bilirubin (pmol/L)	9.92 ± 1.294	10.72 ± 8.356	8.88 ± 3.439	0.431	0.245	0.292	0.913
Cholesterol (CHOL), mmol/L	4.852 ± 1.006 a	5.2 ± 1.178 b	4.97 ± 1.187 a	0.037	0.195	0.068	0.930
High-density lipoprotein cholesterol (HDL-C), mmol/L	1.894 ± 0.272	1.91 ± 0.233	1.61 ± 0.255	0.235	0.069	0.071	0.333
Low-density lipoprotein cholesterol (LDL-C), mmol/L	2.984 ± 0.853 a	3.19 ± 0.919 b	3.29 ± 0.994 b	0.016	0.032	0.073	0.919
Triglycerides (TG), mmol/L	1.908 ± 0.096 b	1.79 ± 0.152 b	1.4 ± 0.079 a	0.248	0.018	0.006	0.417
Glucose (GLU), nmol/L	6.94 ± 0.462	7.14 ± 1.122	6.74 ± 0.607	0.326	0.07	0.155	0.827
Triiodothyronine (T3), nmol/L	2.462 ± 0.165 b	2.35 ± 0.245 b	2.2 ± 0.250 a	0.066	0.017	0.074	0.410
Thyroxine (T4), μ d/L	4.54 ± 0.503 b	3.8 ± 0.367 a	4.1 ± 0.648 a	0.007	0.016	0.103	0.288

(i) basal diet (C-I – control group), (ii) basal diet with extruded-fermented wheat bran added (TG-II), (iii) basal diet with dried sugar beet pulp added (TG-III). ^a, b^Different letters indicate differences among treatments (*p* ≤ 0.05).

A significantly lower (on average, by 2 times) TSH concentration in blood of the TG-III group was found, and the treatment was significant factor on TSH concentration in piglets' blood (*p* = 0.002). In animals, the blood concentrations of thyroid hormones are extremely variable (24). Thyroid hormones may be affected by other nutritional and metabolic facts like levels of zinc, selenium and/or iodine, their supplementation. Also in pigs, there seems to be relationship between energy metabolism and thyroid hormones (24). In TG-II group, higher concentration of ALT was found, in comparison with the C-I group (on average, 15.9% higher). The opposite tendencies were found for the AP concentration, and the highest concentration was detected in C-I group blood samples (on average, 15.8% higher than that in TG-II group). Although the ALT results were in line with the normal range for pigs (25, 26), ALT is a serum marker of liver damage, and its concentration could increase in response to cell membrane damage (27). The high concentrations of AST and ALP in serum during the first week after birth indicated the neonatal pig liver and gut were not fully formed and mature, exhibited by higher membrane permeability, whereas the levels of AST and AP in serum were reduced with the development of piglets and their organs. The highest CHOL concentration was found in TG-II group blood samples. The total serum cholesterol level is an index of lipometabolic status, which includes the free and bound forms of HDL. However, higher concentration of LDL-C in the blood of both treated groups was found (on average, 33.0% higher, in comparison with the C-I group). The TG concentrations were similar in blood samples of the C-I and TG-II groups and 32.1% lower, on average, in blood of the TG-III group, in comparison with C-I and TG-II group samples. Evidence suggests that dietary fiber (DF) binds to bile acids in the small intestine, which makes them less likely to enter the body, decreases intestinal lipid absorption and subsequently reduces the level of cholesterol in the blood (28). Other suggested mechanisms include synthesis by products of bacteria fermentation [production of short-chain fatty acids (SCFAs) such as acetate, butyrate, propionate] (29). SCFAs prevent from intestinal diseases, promote proliferation of intestinal tissue, the absorption of minerals and reduce the level of cholesterol in the blood (30). The hypocholesterolemic effects of DF vary depending on the type and level of fiber fed and it differentially modifies dietary energy absorption, animal's gender also alters the hypocholesterolemic responses to DF. The lowest T4 concentration was found in blood samples of the TG-II and TG-III groups. T4 plays a role in lipid metabolism. This suggests that the increased cholesterol concentrations in piglets might have been caused at least partially by decreased plasma concentrations of T4. The inclusion of dietary fiber (WB and sugar beet pulp are sources of different fiber compounds) can alter the gut microbiota and induce changes in plasma hormone and metabolite concentrations. It can be concluded from the results that all tested creep compound feed compositions were safe and did not impair animal metabolism, since all the values are in the normal range for pigs.

### Fecal pH, dry matter, and color coordinates

Fecal pH, dry matter, and color coordinates are shown in Table 3. In a comparison of fecal pH before the experiment, the mean pH was 6.78 in all tested groups. However, at the end of the experiment, feces of the TG-II group showed a significantly higher pH, in comparison with that of the C-I and TG-III groups (on average, 6.92% higher). However, piglets blood LDL showed a strong negative correlation with fecal pH (*r* = −0.722, *p* = 0.028). Significant differences in fecal dry matter at the beginning of the experiment were not found; but, at the end of the experiment, the highest dry matter content was in feces of the TG-II group (48.72%), and in feces of the C-I and TG-III groups, dry matter was lower by 55.8 and 12.1%, respectively. ANOVA indicated a significant interaction effect of treatment duration and creep compound feed composition on fecal dry matter in piglets (*p* = 0.009). Although, at the beginning of the experiment, TG-III group fecal samples showed the lowest textural hardness, at the end of the experiment, significant differences were not found. Correlations between fecal microbiological and physicochemical parameters and their significance are given in Supplementary material 2. A moderate negative correlation between pH and TCM was established (*r* = 0.501, *p* = 0.034) (Supplementary material 2). Positive moderate correlations between fecal textural hardness and LAB as well as Y/F were found (*r* = 0.475, *p* = 0.046; *r* = 0.567, *p* = 0.014, respectively); furthermore, a strong moderate correlation between fecal textural hardness and TCM was established (*r* = 0.710, *p* = 0.001). These correlations could be explained by the predominance of organic acid producers in TCM; for this reason, a higher TCM was related to a reduction in fecal pH. Also, increasing the LAB and Y/F counts in piglets' feces increased the fecal textural hardness. Usually, an increased LAB count in the digestive tract is associated with a decreased risk of diarrhea. In addition, due to the symbiotic relationship between Y/F and LAB in the same substrate, the avoidance of Y/F growth in feces is very complicated.

**Table 3:** The pH, dry matter and color coordinates of piglet feces.

	**Day**	**C-I**	**TG-II**	**TG-III**	***p*** **day × treatment interaction**
pH	1	6.61 ± 0.82	6.75 ± 0.45	6.98 ± 0.31^B^	0.562
	21	6.64 ± 0.56^a^	7.01 ± 0.77^b^	6.41 ± 0.94^A^^a^	
Dry matter (%)	1	41.21 ± 8.42^B^	40.32 ± 4.74^A^	39.43 ± 5.71	0.009
	21	21.52 ± 3.07^A^^a^	48.72 ± 8.62^B^^c^	42.83 ± 8.35^b^	
Texture (mJ)	1	0.200 ± 0.038^B^^b^	0.200 ± 0.035^B^^b^	0.175 ± 0.029^B^^a^	0.665
	21	0.100 ± 0.023^A^^a^	0.100 ± 0.016^A^^a^	0.100 ± 0.012^A^^a^	
**Color coordinates, NBS**					
L*	1	34.62 ± 3.72^A^	35.99 ± 2.89^A^	34.2 ± 3.73	0.348
	21	41.17 ± 5.22^B^^b^	40.85 ± 5.54^B^^b^	33.7 ± 3.43^a^	
a*	1	0.200 ± 0.029^A^^b^	0.800 ± 0.137^B^^c^	0.010 ± 0.001^B^^a^	<0.001
	21	1.27 ± 0.17^B^^b^	−1.46 ± 0.234^A^^a^	−1.51 ± 0.221^A^^a^	
b*	1	9.20 ± 1.42^A^^b^	6.78 ± 1.06^A^^a^	6.88 ± 1.06^A^^a^	0.242
	21	12.27 ± 2.12^B^^c^	11.26 ± 2.46^B^^b^	7.85 ± 1.65^B^^a^	

(i) basal diet (C-I – control group), (ii) basal diet with extruded-fermented wheat bran added (TG-II), (iii) basal diet with dried sugar beet pulp added (TG-III). The data are presented as the mean ± standard error (*n* = 40/group). 1 – at the beginning of the experiment; 21 – at the end of the experiment.; L^*^, lightness; a^*^, redness or -a^*^, greenness; b^*^, yellowness or -b^*^, blueness; NBS, National Bureau of Standards units; Treat. Int., treatment interaction.

A, B Different capitals indicate significant time-related differences (*p* ≤ 0.05).

a, b Different letters indicate differences among treatments (*p* ≤ 0.05).

In a comparison of fecal color coordinates, at the beginning of the experiment, significant differences between the lightness (L^*^) coordinates were not found; however, at the end of the experiment, C-I and TG-II group samples showed the highest lightness (on average, 41.01 NBS). At the beginning of the experiment, significant differences between the redness and (or) greenness (a^*^ and -a^*^, respectively) coordinates were found, whereas at the end of the experiment, the feces of both treated 'showed similar greenness values (on average, −1.49 NBS), and the C-I group samples showed significantly higher redness values (1.27 NBS). In a comparison of yellowness (b^*^), at the beginning of the experiment, significantly higher b^*^ values were found in C-I group samples, and TG-II and TG-III group samples showed, on average, 25.8% lower b^*^ values. In a comparison of b^*^ values at the end of the experiment, different tendencies were established: TG-III group feces showed the lowest b^*^ (7.85 NBS), and the b^*^ values in C-I and TG-II group feces were, on average, 56.5 and 43.4% higher, respectively.

The consistency, color, odor and volume of the stools are characteristics of certain disease agents. Usually, colibacillosis is associated with alkaline pH (8 and higher), because the feces are rich in bicarbonate. A fecal pH 7 and lower could be associated with atrophic enteritis and malabsorptive diarrhea. Color characteristics are seldom used; however, they could be associated with blood residues in the feces of piglets (<5 days old) suffering from *C. perfringens* type-C enteritis.

Also, it should be pointed out, that there were no cases of diarrhea in any group at the end of the experiment. From this point of view, the differences in fecal parameters could be associated with dietary component variability, that lead to products of varying digestibility and variations in color and pH.

### Volatile compound profiles of piglet feces

**Figure 2:**
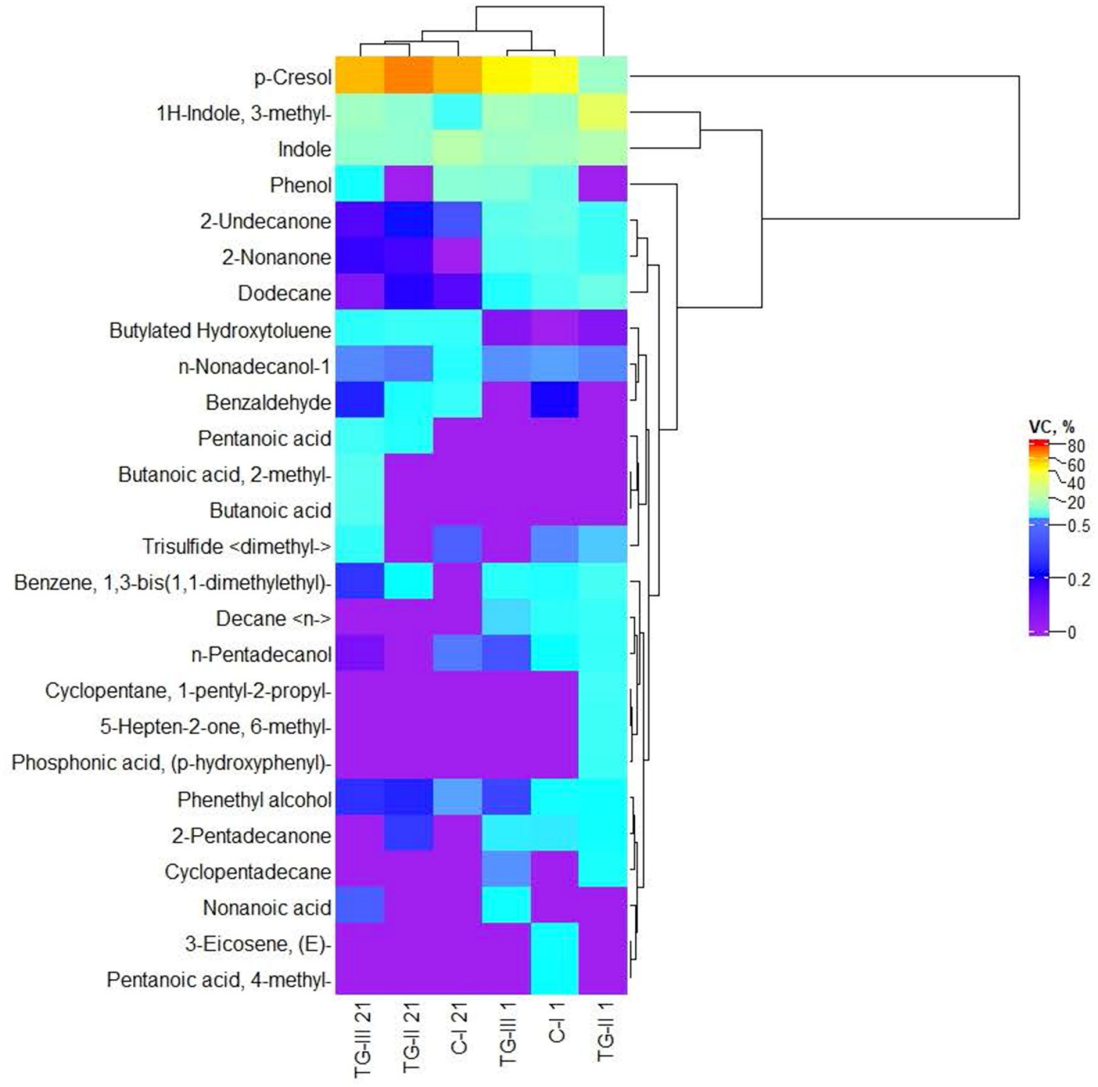
Volatile compound (VC) profile of piglet feces [(i) basal diet (C-I – control group), (ii) basal diet with extruded-fermented wheat bran added (TG-II), (iii) basal diet with dried sugar beet pulp (TG-III)].

Volatile compounds (VC) of which the content was higher than 1% in at least one piglet fecal sample are shown in Figure 2. The whole VC profile is given in Supplementary material 3. Volatile compound profile of piglet feces. The predominant VC in piglet feces were p-cresol; indole and 1H-indole, 3-methyl. Butanoic acid and butanoic acid, 2-methyl- were found only in TG-III group feces at the end of the experiment. Although significant differences in the immunoglobulin concentration between blood samples of different groups of piglets were not found, blood IgA and IgM concentrations showed a strong positive correlation with the fecal volatile compound butanoic acid (*r* = 0.673, *p* = 0.47, and *r* = 0.670, *p* = 0.048, respectively). Pentanoic acid was detected in the feces of both treated groups (TG-II and TG-III) at the end of the experiment. In all the tested groups (at the beginning and end of the experiment), phenethyl alcohol; dodecane; 2-undecanone and n-nonadecanol-1 were found. Pentanoic acid, 4-methyl- and 3-eicosene, (E)- were detected only in the fecal VC profile of the C-I group at the beginning of the experiment, whereas phosphonic acid, (p-hydroxyphenyl)-; 5-hepten-2-one, 6-methyl- and cyclopentane, 1-pentyl-2-propyl- were detected only in the fecal VC profile of the TG-II group at the beginning of the experiment. Decane < n-> was found in the fecal VC profiles of all the groups at the beginning of the experiment. At the beginning of the experiment, 2-nonanone was found in the fecal VC profiles of all the tested groups; however, at the end of the experiment, 2-nonanone did not remain in C-I group feces, and in TG-II and TG-III group feces, its content was reduced by, on average, 15 and 21 times, respectively. Nonanoic acid was detected only in the TG-III group (at the beginning and end of the experiment). Cyclopentadecane was found only at the beginning of the experiment and only in the two treated groups. Butylated hydroxytoluene was found in lower quantities at the beginning of the experiment and was not detected in the C-I group; however, at the end of the experiment, this VC was found in samples from all tested piglets. At the beginning of the experiment, benzene, 1,3-bis(1,1-dimethylethyl)-; n-pentadecanol and 2-pentadecanone were found in all the tested groups; however, at the end of the experiment, their content was reduced and (or) they were not identified in the whole VC profile. The opposite tendencies were found for benzaldehyde: at the beginning of the experiment, this VC was detected only in C-I group feces but was found in the feces of all tested groups at the end of the experiment. Trisulfide < dimethyl-> was found at the beginning of the experiment in feces of the C-I and TG-II groups; however, at the end of the experiment, it was found in feces of the C-I and TG-III groups but not in the TG-II group. Phenol was found in feces of the C-I and TG-III groups at the beginning and end of the experiment. However, different tendencies were found for changes in the phenol content: at the end of the experiment, the quantity of this VC was increased in feces of the C-I group and reduced in that of the TG-III group.

Data on the fecal VC profile of new-born piglets are scarce. Pentanoic acid, 3-methylbutanoic acid, 4-methylphenol, 3-methylindole and indole have been reported in growing pig feces and in manure, slurry, air and dust taken from piggeries (31). Changes in the parameters of new-born piglet feces, including the VC profile, could also be related to their coprophagy, which also helps to prevent anemia in piglets (32).

### Microbiological parameters of fecal samples

Microbiological parameters of the piglets' fecal samples are given in Table 4. In a comparison of the total counts of enterobacteria (TCE) at the beginning of the experiment, the lowest TCE was found in feces of the C-I group; however, at the end of the experiment, the lowest TCE was established in feces of the TG-II group. In feces of the C-I and TG-III groups, TCE was on average 7.2% higher. Usually, *E. coli* lives in symbiosis with its host (33); however, virulent strains can cause diarrhea (33). It was reported that high levels of contamination with enteric bacteria could be a risk factor for the vectorization of these strains (34). Also, T4 concentration in piglets' blood showed very strong positive correlations with TCE and Y/F counts in feces (*r* = 0.991, *p* = 0.0001 and *r* = 0.929, *p* = 0.0001, respectively). In a comparison of the LAB count in piglet feces, significant differences were not found at the beginning of the experiment; however, at the end of the experiment, significantly higher LAB counts were found in both treated groups, in comparison with the C-I group (on average, 22.4 and 10.8% higher in TG-II and TG-III, respectively). ANOVA indicated that the interaction of the duration of treatment and differences in the diet is a significant factor in the LAB count in piglets' feces (*p* = 0.014). An increased population of LAB strains along with the *Bacteroides* genus might contribute to improvements in digestive tract health. More intensive growth of beneficial microorganisms could lead to suppression of pathogen growth (35). In this study, a correlation between LAB and TCE counts in piglets' feces was not found; however, between TCE and TCM, a moderate positive correlation was established (*r* = 0.723, *p* = 0.001). At the beginning of the experiment, a higher total microorganism count (TCM) in the feces of both treated groups was found; however, after 21 days, TCM was the lowest in feces of the TG-III group (on average, 7.8% lower). Piglets blood LDL showed a strong positive correlation with TCM in piglet feces (*r* = 0.750, *p* = 0.020). Significant differences in *Enterococcus faecalis* counts at the end of the experiment in piglets' feces were not found. However, strong positive correlations were established between the piglets' blood TG concentration and *Enterococcus faecalis* as well as Y/F in feces (*r* = 0.746, *p* = 0.021 and *r* = 0.796, *p* = 0.037, respectively). *Enterococcus faecalis* is described as a gut commensal and usually does not cause clinical issues (36). However, this strain can form biofilms and can be 1,000-fold more resistant to antibiotics than planktonic cells (37).

**Table 4:** Microbiological parameters of piglets' fecal samples.

**Microorganism count, log_10_ CFU/g**	**Day**	**C-I**	**TG-II**	**TG-III**	***p*** **day × treatment interaction**
TCE	1	7.17 ± 0.24^a^	7.73 ± 0.368^B^^b^	8.03 ± 0.304 ^B^^c^	0.247
	21	7.31 ± 0.318^b^	6.7 ± 0.339^A^^a^	7.12 ± 1.365^A^^b^	
LAB	1	5.7 ± 0.099 ^A^	6.17 ± 0.007^A^	5.97 ± 0.707 ^A^	0.014
	21	6.11 ± 0.12^B^^a^	7.87 ± 0.106^B^^c^	6.85 ± 0.304^B^^b^	
TCM	1	7.8 ± 0.361^A^^a^	8.79 ± 0.127^B^^b^	8.62 ± 0.919^B^^b^	0.072
	21	8.5 ± 0.523^B^^b^	8.26 ± 0.29^A^^b^	7.73 ± 0.742^A^^a^	
*Enterococcus faecalis*	1	3.52 ± 0.339^A^^b^	6.09 ± 1.266^B^^c^	3.23 ± 0.368^A^^a^	0.238
	21	5.27 ± 2.362^B^	5.45 ± 1.867^A^	5.26 ± 0.983^B^	
Y/F	1	5.32 ± 0.276^A^^a^	5.98 ± 0.233^b^	5.02 ± 1.223^B^^a^	0.275
	21	5.58 ± 0.417^B^^b^	6.12 ± 0.856^c^	4.12 ± 0.127^A^^a^	

(i) basal diet (C-I – control group), (ii) basal diet with extruded-fermented wheat bran added (TG-II), (iii) basal diet with dried sugar beet pulp added (TG-III). The data are presented as the mean ± standard error (*n* = 40/group). 1 – at the beginning of the experiment; 21 – at the end of the experiment. CFU, colony-forming units; LAB, lactic acid bacteria count; TCE, total count of enterobacteria; TCM, total count of aerobic and facultative anaerobic microorganisms; Y/F, yeast/fungi.;

A, B Different capital letters indicate significant time-related differences (*p* ≤ 0.05).

a, b Different letters indicate differences among treatments (*p* ≤ 0.05).

The differences between yeast/fungi (Y/F) counts were established, and, at the beginning and end of the experiment, the highest count of Y/F was found in piglet feces of the TG-II group (on average, 6.05 log_10_ CFU/g). Also, a strong positive correlation between the TSH concentration in blood and the Y/M count in feces was found (*r* = 0.742, *p* = 0.022). In addition, piglets blood ALT showed a very strong positive correlation with TCE in the feces of piglets (*r* = 0.976, *p* = 0.0001), and both blood ALT and ALP showed very strong positive correlations with the Y/F count in fecal samples (*r* = 0.936, *p* = 0.0001 and *r* = 0.831, *p* = 0.006, respectively). The CHOL concentration in blood samples showed a strong positive correlation with the TCE and Y/F counts in piglet feces (*r* = 0.769, *p* = 0.015 and *r* = 0.730, *p* = 0.026, respectively). Synergistic interactions between LAB and yeast have been reported (38); however, most molds are suppressed by LAB action (39). Fungi could produce allergenic spores as well as fungal metabolites (mycotoxins), which pose essential health hazards. For this reason, it is very important to avoid their high numbers in farm environments and reduce fungal counts in the digestive tracts of farm animals.

Also, in our study, some correlations between the VC in feces and microbiological parameters were found. Negative moderate correlations of butyric acid <2-methyl-> and pentanoic acid, 4-methyl- with TCE were established (*r* = −0.476, *p* = 0.046 and *r* = −0.479, *p* = 0.044, respectively) (Supplementary material 2). Trisulfide < dimethyl-> showed a moderate negative correlation with fecal pH (*r* = −0.527, *p* = 0.025). By contrast, p-cresol and butylated hydroxytoluene showed moderate positive correlations with fecal pH (*r* = 0.560, *p* = 0.016 and *r* = 0.547, *p* = 0.019, respectively). 3-Eicosene, (E)- showed a moderate negative correlation with TCE in piglet feces (*r* = −0.477, *p* = 0.046). n-Nonadecanol-1 showed a positive moderate correlation with the LAB count in piglet feces (*r* = 0.534, *p* = 0.022).

Finally, broader research should be performed to indicate individual compounds that could be related to specific health conditions; however, the results obtained are very promising, because parameters with which correlations were established are well known, and the influence of the latter on piglet health could be interpreted.

### Microbial profiles in pig feces

**Figure 3:**
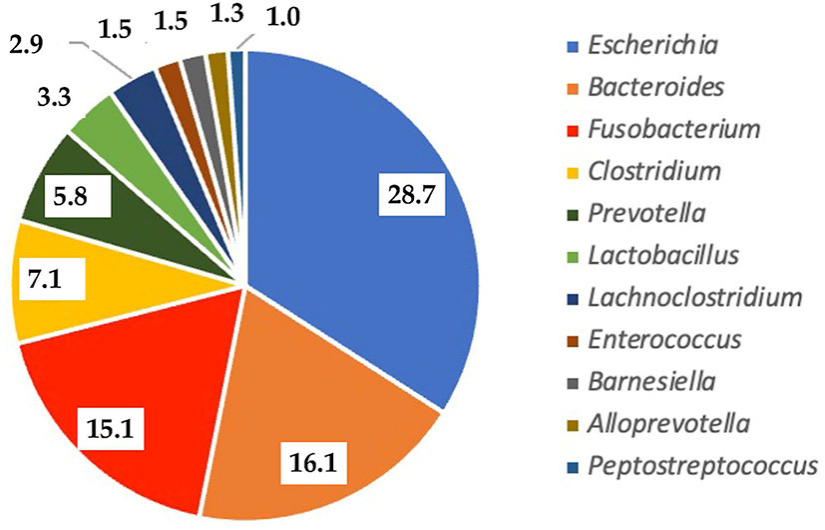
Prevalence of bacterial genera in the gut of piglets before the experiment.

Microbial profiles in piglet feces before the experiment are presented in Figure 3. The predominant genus was *Escherichia*, for which the prevalence among all detected bacteria was 28.7%. Other prevalent genera included *Bacteroides* (16.1%), *Fusobacterium* (15.1%), *Clostridium* (7.1%) and *Prevotella* (5.8%). The less abundant genera were *Lactobacillus, Lachnoclostridium, Enterococcus, Alloprevotella* and *Peptostreptococcus*. Although microbial profiles in piglets can differ depending on the circumstances, the abovementioned genera are frequently found as the normal microbiota in the early life stages of piglets (40, 41). Although *Escherichia* are known as pathogens which can cause diseases in new-born piglets, their high prevalence in pigs does not indicate infection, as *E. coli*, which is the most prevalent species among *Escherichia* is highly variable and usually has no enterotoxigenic properties. This study also demonstrates that *Escherichia* in new-born piglets usually is not associated with disease but is part of the transient microbiota in pigs at a very young age.

**Figure 4:**
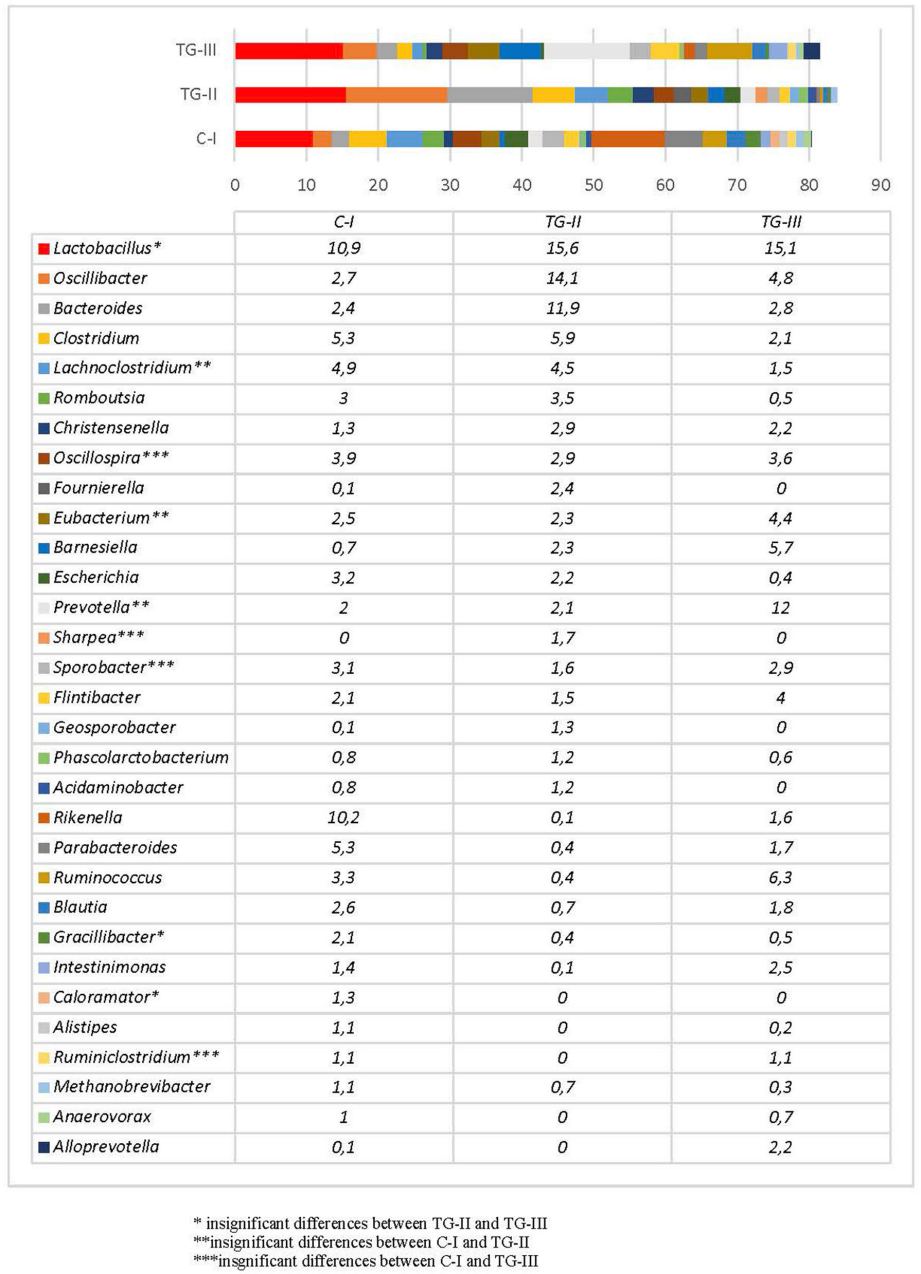
Bacterial composition differences (%) between the animal groups at the end of the experiment [The most prevalent genera (prevalence of at least 1% out of the total amount of bacteria) are presented. (i) Basal diet (C-I – control group), (ii) basal diet with extruded-fermented wheat bran added (TG-II), (iii) basal diet with dried sugar beet pulp added (TG-III)] (* insignificant results between TG-II and TG-III, **insignificant results between C-I and TG-II, ***insignificant results between C-I and TG-III).

**Figure 5:**
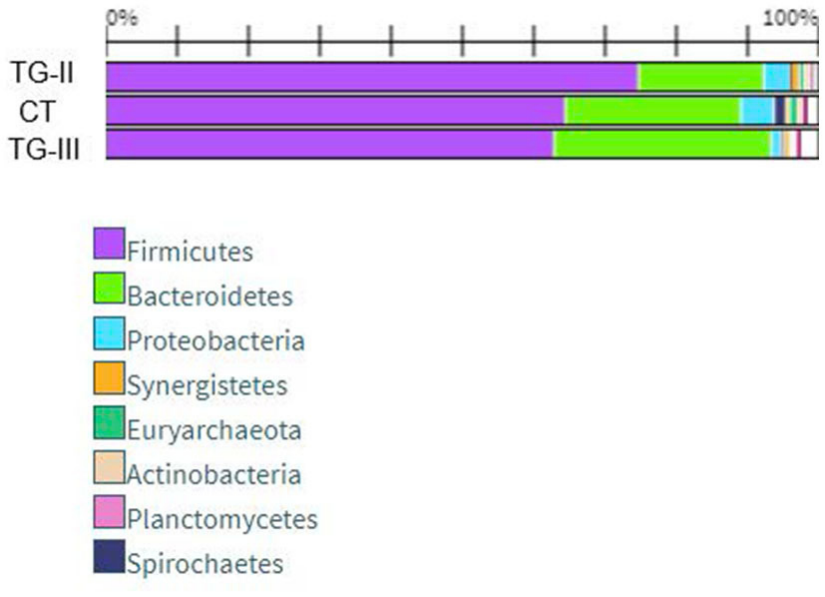
The data about bacterial genera prevalence in different groups of piglets.

Bacterial profiles in pig feces of all the groups at the end of the experiment are presented in Figure 4, 5. At the end of the experiment Firmicutes was the most predominant phylum with the prevalence of more than 60% in all groups of piglets (Figure 4). The second most prevalent phylum was Bacteroidetes which amount varied from 17.6% in TG-II to 30.5% in TH-III. Proteobacteria and other phyla amounted a small part of microbiome. As phyla contain different bacterial genera and species it is complicated to determine the influence of differences in microbial profiles at phylum level between the groups. Figure 5 contains the data about bacterial genera prevalence in different groups of piglets. More detail prevalence of bacterial diversity are presented in Supplementary materials 1–4. There is an evidence that intestinal microbiota impact host's biological activities, including digestion of indigestible feed components, energy harvest, and immunity (42). In this study the highest indices of weight gain were observed in TG-III and the lowest in C-I. The most prevalent genera in TC-III were *Lactobacillus, Ruminococcus*, and *Barnesiella*. There are many studies which demonstrated that different species of *Lactobacillus* were associated with the growth improvement of piglets and promoting the development of small intestinal villi (43). Reduced numbers of *Ruminococcus* can be associated with diarrhea in suckling piglets therefore, this genus also may have association with animal body weight. The resent study also detected significant relation between *Barnesiella* and *Lactobacillus* to the growth performance in Yorkshire pigs (44). *Lactobacillus* also induces porcine host defense peptides and therefore improving immunity (45).

At the end of the experiment, a significant reduction of *Escherichia* in the feces of pigs was detected, compared with that of new-born piglets. This may be explained by microbiota composition changes during different growth periods as *Escherichia* usually presents in high numbers only at early age and normally decreases after weaning (46). The most prevalent genus in all groups was *Lactobacillus*, although the number of species of this genus differed significantly between C-I and the experimental groups (*p* < 0.05). The amount of *Lactobacillus* was higher in the experimental animal groups, whereas there were no statistical differences between groups TG-II and TG-III (*p* = 0.14). The proportions of bacteria of other genera depended on the group. In the C-I group, the most prevalent genera were *Rikenella, Clostridium, Lachnosclostridium*, and *Parabacteroides*, with a total prevalence of 25.7%. In TG-II, the most prevalent were *Oscillibacter, Bacteroides, Clostridium*, and *Lachnoclostridium*, with a total prevalence of 36.4%, and in the TG-III group, *Prevotella, Ruminococcus, Barnesiella*, and *Oscillibacter*, with a total prevalence of 28.8%. The data on these and other bacterial genera detected in separate groups of pigs are presented in Figure 5. The rest of the genera included bacteria often mentioned as the core microbiota in vertebrate animals. Although to date there are no clear data on the role of all microorganisms detected in this study, some knowledge about certain genera exists. For example, *Prevotella* is known to be associated with improvements in the average daily gain and feed conversion ratio in weaned-off pigs (47), while *Ruminococcus* spp. are known as preventive bacteria that reduce the possibility of postweaning diarrhea by producing butyrate, which regulates jejunal homeostasis (48). The amount of *Prevotella* in this study was much higher in the TG-III group, compared with the C-I and TG-II (*p* < 0.05) groups, and it is probably associated with the high carbohydrate content of sugar beet pulp. Piglet feces of this group also contained higher numbers of *Ruminococcus* than the C-I and TG-II groups (*p* < 0.05). *Oscillibacter*, which was the second most prevalent genus after *Lactobacillus* spp. in TG-II, belongs to the order *Clostridiales* and was previously isolated from cows, humans and clams. Regarding a possible biological function of this genus in the gastrointestinal tract, *Oscillibacter* was reported as a probiotic because of the production of anti-inflammatory metabolites (49). Recent results demonstrated that *Oscillibacter* in pigs was associated with feed efficiency (50). Pigs of the TG-II group also had large numbers of *Bacteroides* and *Clostridium*, which are members of the core microbiome in the porcine gut. Pigs in the C-I group had a high prevalence of *Rikenella*—a bacterial genus that is poorly described in association with pigs. It is known as a producer of short-chain fatty acids (51), which are the main energy source of colonocytes and therefore have a positive effect on gastrointestinal health. Other prevalent genera in this group were *Parabacteroides, Clostridium*, and *Lachnoclostridium*, i.e., the normal core microbiota of the porcine gastrointestinal tract. Taking into account the microbial profiles, an increase in beneficial microbiota is visible, particularly of *Lactobacillus* in both experimental groups, whereas different supplementation schemes resulted in different microbial compositions, although both of them had a positive influence: addition of sugar beet pulp resulted in higher numbers of *Prevotella* and *Ruminococcus*, while addition of extruded and fermented WB resulted in increased numbers of *Oscillibacter, Bacteroides*, and *Clostridium*.

## Conclusions

All the tested CCF formulations were safe and did not impair animal metabolism. In addition, the used treatment reduced FCR, increased viable LAB counts in piglet feces, and resulted in better desirable microbial compositions in pigs' gut. Finally, the data suggest that appropriate feed composition can be used for bacterial profile regulation with the aim of supporting animal health and feed efficiency. Also, significant correlations between individual fecal VC and microbiological parameters, and between VC and pH found in this study shed new light on the search for possible chemical markers, as the latter could be used for non-invasive evaluation of piglet health status.

## Data availability statement

The original contributions presented in the study are included in the article/Supplementary material, further inquiries can be directed to the corresponding author.

## Ethics statement

All animal procedures were conducted according to the EU Directive of the European Parliament and of Council from September 22, 2010 (13) on the protection of animals used for scientific purposes and Requirements for the Keeping, Maintenance, and Use of Animals Intended for Science and Education Purposes, approved by the order of the Lithuanian Director of the State Food and Veterinary Service (14). The research was carried out in accordance with both the Republic of Lithuania Act (November 6, 1997) regulating animal care and maintenance and its subsequent legal amendment (Act 8-500) (15).

## Author contributions

The LSMU group conceived the study, participated in its design and coordination, participated in interpretation of the findings, drafted, and reviewed the manuscript. The KTU group participated in the animal studies and interpretation of the findings in the manuscript. The BIOR group developed the methods for feed and biological samples physico-chemical analysis. All authors read and approved the final manuscript.

## Conflict of interest

The authors declare that the research was conducted in the absence of any commercial or financial relationships that could be construed as a potential conflict of interest.

## Publisher's note

All claims expressed in this article are solely those of the authors and do not necessarily represent those of their affiliated organizations, or those of the publisher, the editors and the reviewers. Any product that may be evaluated in this article, or claim that may be made by its manufacturer, is not guaranteed or endorsed by the publisher.

## Supplementary material

Supplementary material 1 Physicochemical, antimicrobial, and antifungal characteristics of the W_ex130/screwspeed25Lpa_.

Supplementary material 2 Correlations between fecal microbiological and physicochemical parameters.

Supplementary material 3 Correlations of fecal microbiological and physicochemical parameters with volatile compounds and their significance.
