# Supplementary material for: Different creep compound feed formulations for new born piglets: influence on growth performance and health parameters
Source: Front Vet Sci. 2022 Aug 29;9:971783. doi: 10.3389/fvets.2022.971783 (PMC9465008; doi:10.3389/fvets.2022.971783)
Supplement: Supplementary material 1 — Physicochemical, antimicrobial, and antifungal characteristics of the Wex130/screwspeed25Lpa. [file Data_Sheet_1.PDF]

Genus Piglets before experiment 22188	Number of reads	Relative abundance
Escherichia	6368	28.7%
Bacteroides	3571	16.09%
Fusobacterium	3346	15.08%
Clostridium	1660	7.48%
Prevotella	1290	5.81%
Lactobacillus	741	3.33%
Lachnoclostridium	650	2.92%
unclassified Fusobacteria	390	1.75%
Unclassified	337	1.51%
Enterococcus	332	1.49%
Barnesiella	328	1.47%
Alloprevotella	285	1.28%
Peptostreptococcus	227	1.02%
Veillonella	198	0.89%
Ruminococcus	197	0.88%
Blautia	183	0.82%
Parabacteroides	160	0.72%
Kluyvera	144	0.64%
Tyzzerella	121	0.54%
Flintibacter	112	0.5%
Eubacterium	94	0.42%
Terrisporobacter	74	0.33%
Flavonifractor	73	0.32%
unclassified Pasteurellaceae	73	0.32%
Shigella	71	0.31%
Desulfovibrio	65	0.29%
Butyricimonas	64	0.28%
Clostridioides	55	0.24%
Eisenbergiella	52	0.23%
unclassified Bacteroidales	50	0.22%
Salmonella	44	0.19%
Ruminiclostridium	39	0.17%
Erysipelatoclostridium	38	0.17%
Streptococcus	37	0.16%
Hespellia	35	0.15%
Casaltella	34	0.15%
Phascolarctobacterium	31	0.13%
Actinobacillus	28	0.12%
Collinsella	22	0.09%
Mobilitalea	21	0.09%
Anaerofilum	20	0.09%
Candidatus Soleaferrea	20	0.09%
Staphylococcus	18	0.08%
Intestinimonas	17	0.07%
Peptoniphilus	16	0.07%
Roseburia	16	0.07%
Eggerthella	15	0.06%
Granulicatella	14	0.06%
Dorea	14	0.06%
Sutterella	13	0.05%
Faecalibacterium	13	0.05%

Actinomyces	13	0.05%
Ruthenibacterium	12	0.05%
Tannerella	12	0.05%
Hungatella	11	0.04%
Vibrio	10	0.04%
Oscillospira	10	0.04%
Kosakonia	9	0.04%
unclassified Lachnospiraceae	9	0.04%
Peptococcus	9	0.04%
Photorhabdus	9	0.04%
Citrobacter	8	0.03%
unclassified Gammaproteobacteria	8	0.03%
Anaerocolumna	8	0.03%
Coprococcus	8	0.03%
Butyricicoccus	7	0.03%
Alistipes	7	0.03%
unclassified Porphyromonadaceae	7	0.03%
Acetivibrio	7	0.03%
Anaeromassilibacillus	7	0.03%
Anaerotaenia	6	0.02%
Succiniclasticum	6	0.02%
Erwinia	6	0.02%
Enterobacter	6	0.02%
Mogibacterium	6	0.02%
Anaerostipes	5	0.02%
Bacillus	5	0.02%
Herbinix	5	0.02%
Helicobacter	5	0.02%
Atopobium	5	0.02%
Negativicoccus	5	0.02%
Robinsoniella	5	0.02%
Serratia	4	0.01%
unclassified Prevotellaceae	4	0.01%
Lactonifactor	4	0.01%
Asaccharospora	4	0.01%
Klebsiella	4	0.01%
Gemmiger	4	0.01%
Bifidobacterium	4	0.01%
Haemophilus	4	0.01%
Pseudoalteromonas	4	0.01%
Paraeggerthella	3	0.01%
Holdemanella	3	0.01%
Falcatimonas	3	0.01%
Levyella	3	0.01%
unclassified Clostridia	3	0.01%
Dielma	3	0.01%
Anaerovorax	3	0.01%
Campylobacter	3	0.01%
Pantoea	3	0.01%
Pseudoflavonifractor	3	0.01%
Turicibacter	2	0%
unclassified Enterobacteriaceae	2	0%

Brassicibacter	2	0%
Parvimonas	2	0%
Cetobacterium	2	0%
Desnuesiella	2	0%
Morganella	2	0%
Finegoldia	2	0%
Pseudocitrobacter	2	0%
Anaerobium	2	0%
Paraprevotella	2	0%
Murimonas	2	0%
Plesiomonas	1	0%
Rothia	1	0%
Alkalibaculum	1	0%
unclassified Streptococcaceae	1	0%
Oribacterium	1	0%
Faecalicoccus	1	0%
Salinimonas	1	0%
Fusicatenibacter	1	0%
Hathewaya	1	0%
Marvinbryantia	1	0%
Defluviitalea	1	0%
Porphyromonas	1	0%
Catonella	1	0%
Hydrogenoanaerobacterium	1	0%
unclassified Betaproteobacteria	1	0%
Sodalis	1	0%
Anaerotruncus	1	0%
Romboutsia	1	0%
Pasteurella	1	0%
Saccharofermentans	1	0%
Oceanirhabdus	1	0%
unclassified Lactobacillaceae	1	0%
Lactococcus	1	0%
unclassified Eubacteriaceae	1	0%
Caloranaerobacter	1	0%
Adlercreutzia	1	0%
Paraclostridium	1	0%
unclassified Veillonellaceae	1	0%
Sporosarcina	1	0%
Bilophila	1	0%
Wautersiella	1	0%
Helcococcus	1	0%
Christensenella	1	0%
Pectobacterium	1	0%
Corynebacterium	1	0%
Mageeibacillus	1	0%
Natranaerovirga	1	0%
unclassified Enterococcaceae	1	0%
unclassified Flavobacteriales	1	0%
unclassified Clostridiales	1	0%
unclassified Alphaproteobacteria	1	0%
Thiothrix	1	0%

Acetanaerobacterium	1	0%
Akkermansia	1	0%
Intestinibacter	1	0%
Pseudomonas	1	0%
