# Supplementary material for: Different creep compound feed formulations for new born piglets: influence on growth performance and health parameters
Source: Front Vet Sci. 2022 Aug 29;9:971783. doi: 10.3389/fvets.2022.971783 (PMC9465008; doi:10.3389/fvets.2022.971783)
Supplement: Supplementary material 2 — Correlations between fecal microbiological and physicochemical parameters. [file Data_Sheet_2.PDF]

| Genera C-I group after experiment | Number of reads |
|---|---|
| Lactobacillus | 2614 |
| Rikenella | 2450 |
| Parabacteroides | 1280 |
| Clostridium | 1264 |
| Lachnoclostridium | 1175 |
| Oscillospira | 938 |
| Ruminococcus | 786 |
| Escherichia | 758 |
| Sporobacter | 747 |
| Romboutsia | 729 |
| Oscillibacter | 658 |
| Blautia | 634 |
| Eubacterium | 597 |
| Bacteroides | 586 |
| Gracilibacter | 512 |
| Flintibacter | 494 |
| Unclassified | 485 |
| Prevotella | 478 |
| unclassified Bacteroidales | 433 |
| Intestinimonas | 342 |
| unclassified Spirochaetia | 308 |
| Caloramator | 306 |
| Christensenella | 304 |
| Alistipes | 261 |
| Ruminiclostridium | 260 |
| Methanobrevibacter | 253 |
| Anaerovorax | 241 |
| Dorea | 217 |
| Desulfovibrio | 196 |
| Acidaminobacter | 195 |
| Phascolarctobacterium | 191 |
| Barnesiella | 172 |
| unclassified Erysipelotrichaceae | 159 |
| Butyricimonas | 156 |
| unclassified Clostridiales Family XIII. Incertae Sedis | 143 |
| Pseudoflavonifractor | 106 |
| unclassified Actinobacteria | 94 |
| Treponema | 92 |
| Hespellia | 92 |
| Holdemania | 84 |
| Ruthenibacterium | 80 |
| Desulfotomaculum | 76 |
| Cloacibacillus | 72 |
| Murimonas | 59 |
| Anaerobacterium | 58 |
| unclassified Planctomycetales | 55 |
| Kluyvera | 51 |
| Lutispora | 48 |
| Hungatella | 47 |
| Acetanaerobacterium | 47 |
| Saccharofermentans | 46 |

| | |
|---|---|
| Rarimicrobium | 43 |
| Defluviitalea | 40 |
| Terrisporobacter | 37 |
| Candidatus Soleaferrea | 36 |
| Vallitalea | 35 |
| Papillibacter | 35 |
| Roseburia | 34 |
| unclassified Clostridiales | 33 |
| Falcatimonas | 32 |
| Coprococcus | 31 |
| Holdemanella | 31 |
| Geosporobacter | 31 |
| Turicibacter | 31 |
| Eisenbergiella | 30 |
| unclassified Eubacteriaceae | 30 |
| Candidatus Heliomonas | 30 |
| Bilophila | 30 |
| Paeniclostridium | 30 |
| Denitrobacterium | 29 |
| Streptococcus | 29 |
| unclassified Ruminococcaceae | 28 |
| Thermotalea | 27 |
| Tyzzerella | 27 |
| Caminicella | 25 |
| Fournierella | 24 |
| Robinsoniella | 23 |
| Natranaerovirga | 22 |
| Anaerotaenia | 22 |
| Alloprevotella | 20 |
| Collinsella | 20 |
| unclassified Clostridia | 20 |
| Syntrophococcus | 19 |
| Intestinibacter | 19 |
| Tannerella | 18 |
| Anaeromassilibacillus | 16 |
| Lactonifactor | 16 |
| Caloranaerobacter | 16 |
| Odoribacter | 15 |
| unclassified Peptostreptococcaceae | 15 |
| Porphyromonas | 15 |
| Succiniclasticum | 13 |
| Shigella | 13 |
| Natronincola | 13 |
| unclassified Lachnospiraceae | 13 |
| Rothia | 13 |
| Anaerofilum | 13 |
| Acetivibrio | 13 |
| Fusicatenibacter | 12 |
| Sanguibacteroides | 12 |
| Candidatus Methanoplasma | 12 |
| Asaccharospora | 11 |
| Catabacter | 10 |

| | |
|---|---|
| Vibrio | 10 |
| Dehalobacterium | 9 |
| Alloscardovia | 9 |
| Abyssivirga | 9 |
| Synergistes | 9 |
| unclassified Mollicutes | 9 |
| Gemmiger | 9 |
| unclassified Deltaproteobacteria | 8 |
| Erysipelothrix | 8 |
| Paludibacter | 8 |
| Anaerotruncus | 8 |
| Flavonifractor | 8 |
| unclassified Alphaproteobacteria | 7 |
| Anaerostipes | 7 |
| Butyricicoccus | 7 |
| Erysipelatoclostridium | 7 |
| Enterococcus | 6 |
| Mogibacterium | 6 |
| Actinomyces | 6 |
| Clostridioides | 6 |
| Anaerocolumna | 6 |
| Tindallia | 6 |
| Acetitomaculum | 5 |
| unclassified Clostridiaceae | 5 |
| Photorhabdus | 5 |
| Alkalibacter | 5 |
| Thermanaerovibrio | 5 |
| unclassified Lactobacillaceae | 5 |
| Peptococcus | 5 |
| Alkaliphilus | 5 |
| Faecalibacterium | 5 |
| unclassified Porphyromonadaceae | 5 |
| Peptostreptococcus | 4 |
| Mobilitalea | 4 |
| Cellulosibacter | 4 |
| Caldicoprobacter | 4 |
| Bacillus | 4 |
| Anaerobium | 4 |
| Jonquetella | 4 |
| unclassified Thermoplasmata | 4 |
| Candidatus Methanomethylophilus | 4 |
| Parasutterella | 3 |
| Faecalicoccus | 3 |
| Staphylococcus | 3 |
| Paraprevotella | 3 |
| Oceanirhabdus | 3 |
| Ercella | 3 |
| Acetoanaerobium | 3 |
| Serpentinicella | 3 |
| Bifidobacterium | 3 |
| Gorbachella | 3 |
| Serratia | 3 |

| | |
|---|---|
| Howardella | 3 |
| Enterobacter | 3 |
| Corynebacterium | 3 |
| Lachnoanaerobaculum | 2 |
| Tepidimicrobium | 2 |
| Gabonibacter | 2 |
| Salmonella | 2 |
| Actinomadura | 2 |
| Parasporobacterium | 2 |
| Butyrivibrio | 2 |
| Alkalibaculum | 2 |
| Veillonella | 2 |
| Desnuesiella | 2 |
| Oxobacter | 2 |
| Kosakonia | 2 |
| Adlercreutzia | 2 |
| Macellibacteroides | 2 |
| Dielma | 2 |
| Desulfitobacterium | 2 |
| Olivibacter | 2 |
| unclassified Pasteurellaceae | 2 |
| Solobacterium | 2 |
| Alteromonas | 2 |
| Proteiniborus | 2 |
| Fusobacterium | 2 |
| unclassified Betaproteobacteria | 2 |
| Paraeggerthella | 2 |
| Fonticella | 2 |
| unclassified Veillonellaceae | 2 |
| unclassified Fusobacteria | 1 |
| Herbinix | 1 |
| Geobacter | 1 |
| Anaerofustis | 1 |
| Akkermansia | 1 |
| unclassified Erysipelotrichia | 1 |
| Tepidibacter | 1 |
| Thermoflavimicrobium | 1 |
| Parvibacter | 1 |
| unclassified Rikenellaceae | 1 |
| Enterobacillus | 1 |
| Dethiosulfovibrio | 1 |
| Crassaminicella | 1 |
| Pseudomonas | 1 |
| Prolixibacter | 1 |
| unclassified Victivallaceae | 1 |
| Erwinia | 1 |
| Atopobium | 1 |
| Enorma | 1 |
| Oxalobacter | 1 |
| Anaerorhabdus | 1 |
| unclassified Gammaproteobacteria | 1 |
| Elusimicrobium | 1 |

| | |
|---|---|
| Paenibacillus | 1 |
| Ethanoligenens | 1 |
| Brassicibacter | 1 |
| Citrobacter | 1 |
| Haemophilus | 1 |
| Acidaminococcus | 1 |
| Achromobacter | 1 |
| unclassified Firmicutes | 1 |
| Victivallis | 1 |
| Garciella | 1 |
| Sutterella | 1 |
| Sphaerochaeta | 1 |
| unclassified Spirochaetales | 1 |
| Klebsiella | 1 |
| Terasakiella | 1 |
| Propionispira | 1 |
| unclassified Bacteroidaceae | 1 |
| Streptomyces | 1 |
| Hydrogenoanaerobacterium | 1 |
| Henriciella | 1 |
| Cryptanaerobacter | 1 |
| Petrimonas | 1 |
| Proteocatella | 1 |
| unclassified Cyanobacteria | 1 |
| Pelotomaculum | 1 |
| Lachnospira | 1 |
| Exiguobacterium | 1 |
| Actinobacillus | 1 |
| Ornithinibacillus | 1 |

| **Relative abundance** |
| --- |
| 10.87% |
| 10.19% |
| 5.32% |
| 5.25% |
| 4.88% |
| 3.9% |
| 3.26% |
| 3.15% |
| 3.1% |
| 3.03% |
| 2.73% |
| 2.63% |
| 2.48% |
| 2.43% |
| 2.12% |
| 2.05% |
| 2.01% |
| 1.98% |
| 1.8% |
| 1.42% |
| 1.28% |
| 1.27% |
| 1.26% |
| 1.08% |
| 1.08% |
| 1.05% |
| 1% |
| 0.9% |
| 0.81% |
| 0.81% |
| 0.79% |
| 0.71% |
| 0.66% |
| 0.64% |
| 0.59% |
| 0.44% |
| 0.39% |
| 0.38% |
| 0.38% |
| 0.34% |
| 0.33% |
| 0.31% |
| 0.29% |
| 0.24% |
| 0.24% |
| 0.22% |
| 0.21% |
| 0.19% |
| 0.19% |
| 0.19% |
| 0.19% |

0.17%
0.16%
0.15%
0.14%
0.14%
0.14%
0.14%
0.13%
0.13%
0.12%
0.12%
0.12%
0.12%
0.12%
0.12%
0.12%
0.12%
0.12%
0.12%
0.12%
0.11%
0.11%
0.11%
0.1%
0.09%
0.09%
0.09%
0.09%
0.08%
0.08%
0.08%
0.07%
0.07%
0.07%
0.06%
0.06%
0.06%
0.06%
0.06%
0.06%
0.05%
0.05%
0.05%
0.05%
0.05%
0.05%
0.05%
0.04%
0.04%
0.04%
0.04%
0.04%

0.04%
0.03%
0.03%
0.03%
0.03%
0.03%
0.03%
0.03%
0.03%
0.03%
0.03%
0.03%
0.02%
0.02%
0.02%
0.02%
0.02%
0.02%
0.02%
0.02%
0.02%
0.02%
0.02%
0.02%
0.02%
0.02%
0.02%
0.02%
0.02%
0.02%
0.02%
0.02%
0.01%
0.01%
0.01%
0.01%
0.01%
0.01%
0.01%
0.01%
0.01%
0.01%
0.01%
0.01%
0.01%
0.01%
0.01%
0.01%
0.01%
0.01%
0.01%
0.01%

0.01%
0.01%
0.01%
0%
0%
0%
0%
0%
0%
0%
0%
0%
0%
0%
0%
0%
0%
0%
0%
0%
0%
0%
0%
0%
0%
0%
0%
0%
0%
0%
0%
0%
0%
0%
0%
0%
0%
0%
0%
0%
0%
0%
0%
0%
0%
0%
0%
0%
0%
0%
0%
0%

0%
0%
0%
0%
0%
0%
0%
0%
0%
0%
0%
0%
0%
0%
0%
0%
0%
0%
0%
0%
0%
0%
0%
0%
0%
0%
0%
0%
0%
