# Supplementary material for: Different creep compound feed formulations for new born piglets: influence on growth performance and health parameters
Source: Front Vet Sci. 2022 Aug 29;9:971783. doi: 10.3389/fvets.2022.971783 (PMC9465008; doi:10.3389/fvets.2022.971783)
Supplement: Supplementary material 3 — Correlations of fecal microbiological and physicochemical parameters with volatile compounds and their significance. [file Data_Sheet_3.PDF]

Genera TG-II after experiment	Number of reads
Lactobacillus	3164
Oscillibacter	2853
Bacteroides	2396
Clostridium	1199
Lachnoclostridium	910
Romboutsia	710
Christensenella	579
Oscillospira	578
Fournierella	479
Eubacterium	474
Barnesiella	466
Escherichia	448
Prevotella	434
Sharpea	335
Sporobacter	324
Flintibacter	294
Geosporobacter	266
Phascolarctobacterium	248
Acidaminobacter	246
Unclassified	195
Caloramator	177
Cloacibacillus	164
Enterococcus	157
Ruminiclostridium	156
Methanobrevibacter	140
Blautia	137
Intestinimonas	136
Acetanaerobacterium	113
Veillonella	109
unclassified Bacteroidales	103
unclassified Planctomycetales	95
Parabacteroides	90
Anaerotruncus	89
Ruminococcus	86
Gracilibacter	82
Tyzzerella	80
Turicibacter	68
Ruthenibacterium	57
Campylobacter	55
Collinsella	54
Desulfovibrio	48
Desulfotomaculum	46
unclassified Clostridiales Family XIII. Incertae Sedis	45
Klebsiella	45
Synergistes	43
Caminicella	42
Dorea	37
Pseudoflavonifractor	37
Natranaerovirga	36
Hespellia	36
Erysipelatoclostridium	35

Streptococcus	34
Lutispora	33
Hungatella	29
Kluyvera	26
Intestinibacter	25
Rikenella	25
Terrisporobacter	24
Eisenbergiella	24
Mobilitalea	23
Helicobacter	22
Rothia	21
unclassified Clostridiales	18
Coprococcus	18
Falcatimonas	17
Paeniclostridium	16
Acetivibrio	16
Tindallia	16
unclassified Peptostreptococcaceae	15
Peptococcus	14
Saccharofermentans	14
Olsenella	14
Caloranaerobacter	14
Shigella	14
Thermanaerovibrio	14
unclassified Lachnospiraceae	14
Flavonifractor	14
Asaccharospora	13
Treponema	13
Bifidobacterium	13
Bilophila	13
Candidatus Soleaferrea	13
Eggerthella	11
Denitrobacterium	11
Paraprevotella	11
Photorhabdus	11
Anaeromassilibacillus	10
Catenibacterium	9
unclassified Ruminococcaceae	9
Mahella	9
Bacillus	9
Thermotalea	9
Roseburia	8
Vibrio	8
Kosakonia	8
Faecalicoccus	8
Natronincola	8
Pyramidobacter	7
Tannerella	7
Proteiniborus	7
Pseudoalteromonas	7
Actinobacillus	7
Holdemanella	7

Murimonas	7
Comamonas	6
Peptostreptococcus	6
unclassified Alphaproteobacteria	6
Butyricicoccus	6
Caldicoprobacter	6
Lactococcus	6
unclassified Thermoplasmata	6
Anaerotaenia	6
Butyricimonas	5
Anaerovorax	5
Candidatus Nardonella	5
Catabacter	5
Wukongibacter	5
Herbinix	5
Candidatus Heliomonas	5
Papillibacter	4
Faecalibacterium	4
Thermoflavimicrobium	4
Lactonifactor	4
Porphyromonas	4
Oceanirhabdus	4
Defluviitalea	4
Dehalobacterium	4
Alkalibacter	4
Garciella	4
Anaerostipes	4
Paraeggerthella	4
Brassicibacter	3
Dethiosulfovibrio	3
unclassified Pasteurellaceae	3
Paludibacter	3
unclassified Clostridia	3
Alloprevotella	3
Salmonella	3
Serratia	3
Sanguibacteroides	3
unclassified Actinobacteria	3
Ornithinibacillus	3
Alkaliphilus	3
Clostridioides	3
Parvibacter	3
Staphylococcus	3
Fusobacterium	3
Lachnoanaerobaculum	3
unclassified Betaproteobacteria	3
Actinomyces	3
Enterobacter	2
Hydrogenoanaerobacterium	2
unclassified Spirochaetales	2
Citrobacter	2
unclassified Lactobacillaceae	2

Ethanoligenens	2
Butyrivibrio	2
Ercella	2
Fibrobacter	2
Acetoanaerobium	2
Oligosphaera	2
Atopobium	2
Corynebacterium	2
Vallitalea	2
Anaerocolumna	2
Gabonibacter	2
Sphingobacterium	2
Abyssivirga	2
unclassified Clostridiaceae	2
Alteromonas	2
Proteinivorax	1
Mangroviflexus	1
Caldilinea	1
Victivallis	1
unclassified Eubacteriaceae	1
Sedimentibacter	1
Raoultella	1
Fusicatenibacter	1
unclassified Firmicutes	1
Bariatricus	1
Arcanobacterium	1
Sphaerochaeta	1
Megasphaera	1
Anaerobacterium	1
Gottschalkia	1
Jonquetella	1
Enorma	1
Halomonas	1
Candidatus Methanoplasma	1
Aminobacterium	1
Novibacillus	1
Fonticella	1
Pseudoramibacter	1
Sellimonas	1
unclassified Erysipelotrichaceae	1
Helcococcus	1
Selenomonas	1
Nitrincola	1
Howardella	1
Anaerofilum	1
Prolixibacter	1
Pluralibacter	1
Exiguobacterium	1
Marvinbryantia	1
unclassified Prevotellaceae	1
Shewanella	1
Pectobacterium	1

Pseudomonas	1
Olivibacter	1
Tepidibacter	1
Natranaerobaculum	1
Sphingobium	1
Gordonibacter	1
Robinsoniella	1
Acetatifactor	1
Erysipelothrix	1
Anaerofustis	1
Pantoea	1
Pediococcus	1
Crassaminicella	1
Enterorhabdus	1
Desulfitispora	1
Holdemania	1
Acidaminococcus	1

Relative abundance
15.62%
14.09%
11.83%
5.92%
4.49%
3.5%
2.85%
2.85%
2.36%
2.34%
2.3%
2.21%
2.14%
1.65%
1.6%
1.45%
1.31%
1.22%
1.21%
0.96%
0.87%
0.81%
0.77%
0.77%
0.69%
0.67%
0.67%
0.55%
0.53%
0.5%
0.46%
0.44%
0.43%
0.42%
0.4%
0.39%
0.33%
0.28%
0.27%
0.26%
0.23%
0.22%
0.22%
0.22%
0.21%
0.2%
0.18%
0.18%
0.17%
0.17%
0.17%

0.16%
0.16%
0.14%
0.12%
0.12%
0.12%
0.11%
0.11%
0.11%
0.1%
0.1%
0.08%
0.08%
0.08%
0.07%
0.07%
0.07%
0.07%
0.06%
0.06%
0.06%
0.06%
0.06%
0.06%
0.06%
0.06%
0.06%
0.06%
0.06%
0.06%
0.06%
0.05%
0.05%
0.05%
0.05%
0.04%
0.04%
0.04%
0.04%
0.04%
0.04%
0.03%
0.03%
0.03%
0.03%
0.03%
0.03%
0.03%
0.03%
0.03%
0.03%
0.03%

0.03%
0.02%
0.02%
0.02%
0.02%
0.02%
0.02%
0.02%
0.02%
0.02%
0.02%
0.02%
0.02%
0.02%
0.02%
0.02%
0.01%
0.01%
0.01%
0.01%
0.01%
0.01%
0.01%
0.01%
0.01%
0.01%
0.01%
0.01%
0.01%
0.01%
0.01%
0.01%
0.01%
0.01%
0.01%
0.01%
0.01%
0.01%
0.01%
0.01%
0.01%
0.01%
0.01%
0.01%
0.01%
0.01%
0.01%
0%
0%
0%
0%
0%

0%
0%
0%
0%
0%
0%
0%
0%
0%
0%
0%
0%
0%
0%
0%
0%
0%
0%
0%
0%
0%
0%
0%
0%
0%
0%
0%
0%
0%
0%
0%
0%
0%
0%
0%
0%
0%
0%
0%
0%
0%
0%
0%
0%
0%
0%
0%
0%
0%
0%
0%
0%

0%
0%
0%
0%
0%
0%
0%
0%
0%
0%
0%
0%
0%
0%
0%
0%
0%
