# Supplementary material for: Different creep compound feed formulations for new born piglets: influence on growth performance and health parameters
Source: Front Vet Sci. 2022 Aug 29;9:971783. doi: 10.3389/fvets.2022.971783 (PMC9465008; doi:10.3389/fvets.2022.971783)
Supplement: Supplementary file 4 [file Data_Sheet_4.PDF]

Genera TG-III after experiment	Number of reads
Lactobacillus	2804
Prevotella	2224
Ruminococcus	1171
Barnesiella	1059
Oscillibacter	888
Eubacterium	813
Flintibacter	735
Oscillospira	668
unclassified Bacteroidales	559
Sporobacter	543
Unclassified	531
Bacteroides	514
Intestinimonas	468
Alloprevotella	416
Christensenella	411
Clostridium	398
Blautia	328
Parabacteroides	322
Rikenella	290
Lachnoclostridium	284
Ruminiclostridium	199
Anaerovorax	125
Tannerella	123
Falcatimonas	113
Phascolarctobacterium	113
Pseudoflavonifractor	108
Desulfovibrio	108
Gracilibacter	92
Romboutsia	87
Escherichia	79
Papillibacter	77
Holdemania	69
unclassified Planctomycetales	67
Cloacibacillus	63
unclassified Clostridiales Family XIII. Incertae Sedis	54
Anaerotruncus	54
Candidatus Soleaferrea	54
Dorea	53
Methanobrevibacter	52
unclassified Clostridiales	48
Alistipes	44
unclassified Spirochaetia	44
Paraprevotella	39
Treponema	39
Saccharofermentans	38
Roseburia	36
Pyramidobacter	36
unclassified Lachnospiraceae	33
Porphyromonas	30
Defluviitalea	30
Eisenbergiella	29

Flavonifractor	28
unclassified Ruminococcaceae	28
Hungatella	28
Murimonas	28
Terrisporobacter	27
Turicibacter	26
Veillonella	26
Denitrobacterium	23
unclassified Actinobacteria	22
Ruthenibacterium	21
Faecalicoccus	21
unclassified Cyanobacteria	20
Anaerobacterium	19
unclassified Clostridia	19
Tyzzerella	18
Coprococcus	17
Fusobacterium	17
Acetivibrio	17
Streptococcus	17
Fusicatenibacter	16
Desulfotomaculum	16
Lutispora	16
Bilophila	16
Anaerotaenia	15
Synergistes	15
Anaerostipes	15
Butyricimonas	13
Natranaerovirga	13
Anaeromassilibacillus	13
Rarimicrobium	12
Intestinibacter	11
Anaerocolumna	11
Hespellia	11
Succiniclasticum	11
Fucophilus	11
Acetanaerobacterium	11
Paludibacter	11
Mogibacterium	10
Acidaminobacter	10
Catabacter	10
Holdemanella	10
Brassicibacter	10
Vibrio	10
unclassified Fusobacteria	9
Sphaerochaeta	8
Caloramator	8
Faecalibacterium	8
unclassified Veillonellaceae	7
Collinsella	7
Fournierella	6
Elusimicrobium	6
Sharpea	6

Campylobacter	6
Ethanoligenens	5
Olivibacter	5
Victivallis	5
Erysipelothrix	5
Alkalibacter	5
Herbinix	5
unclassified Eubacteriaceae	4
Mageeibacillus	4
Bifidobacterium	4
Butyricicoccus	4
Peptococcus	4
Howardella	4
unclassified Prevotellaceae	4
Geobacter	4
Caminicella	4
Shigella	4
Mobilitalea	4
unclassified Pasteurellaceae	4
Asaccharospora	4
Rothia	4
unclassified Erysipelotrichaceae	4
Bittarella	4
Butyrivibrio	4
Clostridioides	3
Geosporobacter	3
Abyssivirga	3
Enorma	3
Actinobacillus	3
Vallitalea	3
Actinomadura	3
Kluyvera	3
Pseudoalteromonas	2
Lachnoanaerobaculum	2
Garciella	2
Sanguibacteroides	2
Oceanirhabdus	2
unclassified Clostridiaceae	2
Fonticella	2
unclassified Peptostreptococcaceae	2
Alkalibaculum	2
Lactonifactor	2
Oribacterium	2
Solobacterium	2
Desnuesiella	2
unclassified Porphyromonadaceae	2
Citrobacter	2
Ercella	2
Acetitomaculum	2
Sutterella	2
Acetoanaerobium	2
Alkaliphilus	2

Spirochaeta	2
Enterococcus	2
Desulfitobacterium	2
Candidatus Vestibaculum	2
Oxalobacter	1
Salmonella	1
Lewinella	1
Caloranaerobacter	1
Alteromonas	1
Rhodospirillum	1
Rheinheimera	1
Anaerovibrio	1
Candidatus Heliomonas	1
Dielma	1
Corynebacterium	1
Gemmiger	1
Thermotalea	1
Lacibacterium	1
Anaerocella	1
Oxobacter	1
Parasporobacterium	1
unclassified Thermoplasmata	1
unclassified Bacilli	1
Adlercreutzia	1
Pediococcus	1
Proteinivorax	1
Propionibacterium	1
Ureibacillus	1
Proteiniborus	1
Paraglaciecola	1
Staphylococcus	1
Anaerorhabdus	1
Jonquetella	1
Caldicoprobacter	1
Paeniclostridium	1
Cellulosilyticum	1
Trigonala	1
Thermanaeromonas	1
Gottschalkia	1
unclassified Deltaproteobacteria	1
Petrimonas	1
Desulfonispora	1
Anaerofilum	1
Faecalitalea	1
unclassified Bacteroidia	1
Serpentinicella	1
Erysipelatoclostridium	1
Geoalkalibacter	1
Streptomyces	1
Marvinbryantia	1
Syntrophococcus	1
Dehalobacterium	1

Syntrophomonas	1
Selenomonas	1
Herbaspirillum	1
Agathobacter	1
unclassified Erysipelotrichia	1
Acetatifactor	1
Anaerobium	1
Pleomorphochaeta	1
Robinsoniella	1
Thermanaerovibrio	1

Relative abundance
15.09%
11.96%
6.3%
5.69%
4.77%
4.37%
3.95%
3.59%
3%
2.92%
2.85%
2.76%
2.51%
2.23%
2.21%
2.14%
1.76%
1.73%
1.56%
1.52%
1.07%
0.67%
0.66%
0.6%
0.6%
0.58%
0.58%
0.49%
0.46%
0.42%
0.41%
0.37%
0.36%
0.33%
0.29%
0.29%
0.29%
0.28%
0.27%
0.25%
0.23%
0.23%
0.2%
0.2%
0.2%
0.19%
0.19%
0.17%
0.16%
0.16%
0.15%

0.15%
0.15%
0.15%
0.15%
0.14%
0.13%
0.13%
0.12%
0.11%
0.11%
0.11%
0.1%
0.1%
0.1%
0.09%
0.09%
0.09%
0.09%
0.09%
0.08%
0.08%
0.08%
0.08%
0.08%
0.08%
0.08%
0.06%
0.06%
0.06%
0.06%
0.05%
0.05%
0.05%
0.05%
0.05%
0.05%
0.05%
0.05%
0.05%
0.05%
0.05%
0.05%
0.05%
0.04%
0.04%
0.04%
0.04%
0.03%
0.03%
0.03%
0.03%
0.03%

0.03%
0.02%
0.02%
0.02%
0.02%
0.02%
0.02%
0.02%
0.02%
0.02%
0.02%
0.02%
0.02%
0.02%
0.02%
0.02%
0.02%
0.02%
0.02%
0.02%
0.02%
0.02%
0.02%
0.02%
0.01%
0.01%
0.01%
0.01%
0.01%
0.01%
0.01%
0.01%
0.01%
0.01%
0.01%
0.01%
0.01%
0.01%
0.01%
0.01%
0.01%
0.01%
0.01%
0.01%
0.01%
0.01%
0.01%
0.01%
0.01%
0.01%
0.01%
0.01%

0.01%
0.01%
0.01%
0.01%
0%
0%
0%
0%
0%
0%
0%
0%
0%
0%
0%
0%
0%
0%
0%
0%
0%
0%
0%
0%
0%
0%
0%
0%
0%
0%
0%
0%
0%
0%
0%
0%
0%
0%
0%
0%
0%
0%
0%
0%
0%
0%
0%
0%
0%
0%
0%
0%

0%
0%
0%
0%
0%
0%
0%
0%
0%
0%
